# Differential trajectories of hypometabolism across cognitively-defined Alzheimer’s disease subgroups

**DOI:** 10.1016/j.nicl.2021.102725

**Published:** 2021-06-12

**Authors:** Colin Groot, Shannon L. Risacher, J.Q. Alida Chen, Ellen Dicks, Andrew J. Saykin, Christine L. Mac Donald, Jesse Mez, Emily H. Trittschuh, Shubhabrata Mukherjee, Frederik Barkhof, Philip Scheltens, Wiesje M. van der Flier, Rik Ossenkoppele, Paul K. Crane

**Affiliations:** a Department of Neurology & Alzheimer Center, Amsterdam Neuroscience, Vrije Universiteit Amsterdam, Amsterdam UMC, Amsterdam, The Netherlands; b Indiana University School of Medicine, Indianapolis, IN, USA; c Neurological Surgery, University of Washington, Seattle, WA, USA; d Department of Neurology, Boston University School of Medicine, Boston, MA, USA; e Alzheimer’s Disease Center, Boston University School of Medicine, MA, USA; f Psychiatry & Behavioral Science, University of Washington, Seattle, WA, USA; g Veterans Affairs Puget Sound Health Care System, Geriatric Research, Education, & Clinical Center, Seattle, WA, USA; h Department of Medicine, University of Washington, Seattle, WA, USA; i Department of Radiology and Nuclear Medicine, Amsterdam Neuroscience Vrije Universiteit Amsterdam, Amsterdam UMC, Amsterdam, The Netherlands; j University College London, Institutes of Neurology & Healthcare Engineering, London, United Kingdom; k Epidemiology and Data Science, Vrije Universiteit Amsterdam, Amsterdam UMC, Amsterdam, The Netherlands; l Lund University, Clinical Memory Research Unit, Lund, Sweden

**Keywords:** AD, Alzheimer’s disease, ACT, Adult changes in thought cohort, ADNI, Alzheimer’s disease neuroimaging initiative, APOE, Apolipoprotein E, CSF, Cerebrospinal fluid, DARTEL, Diffeomorphic anatomical registration through exponentiated lie algebra, FDG, Fluorodeoxyglucose, PET, Positron emission tomography, lvPPA, Logopenic variant primary progressive aphasia, MMSE, Mini-mental state examination, SPM, Statistical parametric mapping, MAP, the Rush Memory and Aging Project, ROS, Religious Orders Study, SD, standard deviation, AAL, Automatic anatomical labelling, MTL, medial temporal lobe, PCA, posterior cortical atrophy, TPC, temporoparietal cortex, Alzheimer’s disease, FDG-PET, Heterogeneity, Psychometrics

## Abstract

Disentangling biologically distinct subgroups of Alzheimer’s disease (AD) may facilitate a deeper understanding of the neurobiology underlying clinical heterogeneity. We employed longitudinal [^18^F]FDG-PET standardized uptake value ratios (SUVRs) to map hypometabolism across cognitively-defined AD subgroups. Participants were 384 amyloid-positive individuals with an AD dementia diagnosis from ADNI who had a total of 1028 FDG-scans (mean time between first and last scan: 1.6 ± 1.8 years). These participants were categorized into subgroups on the basis of substantial impairment at time of dementia diagnosis in a specific cognitive domain relative to the average across domains. This approach resulted in groups of AD-Memory (n = 135), AD-Executive (n = 8), AD-Language (n = 22), AD-Visuospatial (n = 44), AD-Multiple Domains (n = 15) and AD-No Domains (for whom no domain showed substantial relative impairment; n = 160). Voxelwise contrasts against controls revealed that all AD-subgroups showed progressive hypometabolism compared to controls across temporoparietal regions at time of AD diagnosis. Voxelwise and regions-of-interest (ROI)-based linear mixed model analyses revealed there were also subgroup-specific hypometabolism patterns and trajectories. The AD-Memory group had more pronounced hypometabolism compared to all other groups in the medial temporal lobe and posterior cingulate, and faster decline in metabolism in the medial temporal lobe compared to AD-Visuospatial. The AD-Language group had pronounced lateral temporal hypometabolism compared to all other groups, and the pattern of metabolism was also more asymmetrical (left < right) than all other groups. The AD-Visuospatial group had faster decline in metabolism in parietal regions compared to all other groups, as well as faster decline in the precuneus compared to AD-Memory and AD-No Domains. Taken together, in addition to a common pattern, cognitively-defined subgroups of people with AD dementia show subgroup-specific hypometabolism patterns, as well as differences in trajectories of metabolism over time. These findings provide support to the notion that cognitively-defined subgroups are biologically distinct.

## Highlights

• Cognitive-subgroups can be identified among individuals with AD dementia.
• Subgroup-specific patterns and longitudinal trajectories of hypometabolism observed.
• Regional hypometabolism matched respective cognitive profiles of subgroups.
• Cognitive-classification yields biologically distinct subgroups.

## 1 Introduction

Alzheimer’s disease (AD) dementia is commonly regarded as an amnestic disorder. However, there is considerable heterogeneity in clinical presentations among individuals with AD, and sometimes non-amnestic impairments are a prominent feature (Crane et al., 2017, Scheltens et al., 2017). Our group has developed and applied a framework that categorizes individual people with AD into cognitively-defined AD-subgroups based on cognitive data by exploiting relative impairments across cognitive domains at time of dementia diagnosis (Crane et al., 2017). Previous examinations have shown that groups with relative impairments in domains other than memory show faster cognitive and functional decline than individuals with substantial relative impairments in memory (Mez et al., 2013b, Mez et al., 2013a). Furthermore, findings in groups of individuals with atypical non-amnestic variants of AD such as logopenic primary progressive aphasia (lvPPA) and posterior cortical atrophy (PCA) (Gorno-Tempini et al., 2011, Crutch et al., 2017) revealed distinct patterns of atrophy at time of dementia diagnosis. These findings collectively suggest that clinical heterogeneity across people with AD is related to differences in the neurobiological substrate.

[^18^F]FDG-PET has long been used to differentiate between neurodegenerative diseases (Rice and Bisdas, 2017, Shivamurthy et al., 2015), and has been an instrumental tool to detect AD patterns of hypometabolism in individuals before they develop AD dementia (Laforce et al., 2018, Rice and Bisdas, 2017, Sala et al., 2020, Tripathi et al., 2014). The typical pattern of hypometabolism in AD spans across temporoparietal and posterior cingulate regions (Laforce et al., 2018), and [^18^F]FDG-PET shows good sensitivity for detecting early AD-related changes (Bloudek et al., 2011, Laforce et al., 2018, Rice and Bisdas, 2017). Previous studies have found associations between clinical symptoms and spatial patterns of hypometabolism (Besson et al., 2015, Laforce et al., 2018, Vanhoutte et al., 2017), indicating that FDG-PET is a good candidate to map trajectories of metabolism associated with differences in the clinical expression of AD.

This paper aims to add to the literature in two ways: First, it investigates whether regional glucose metabolism patterns as measured by FDG-PET at time of AD diagnosis differ across cognitively-defined subgroups. Second, this paper considers longitudinal data, which could supply additional support to the notion that cognitively-defined subgroups have distinct natural histories.

## 2 Materials and methods

### 2.1 Participants

Data for the present study were obtained from the publicly available Alzheimer’s Disease Neuroimaging Initiative (ADNI) cohort. Inclusion criteria for the present study were: i) clinical diagnosis of AD, either at ADNI enrolment (prevalent AD dementia) or at any of the follow-up visits (incident AD dementia), ii) amyloid-positive, as indicated by CSF measures or by amyloid-PET scan findings at time of dementia diagnosis, and iii) availability of at least one FDG-PET with corresponding MRI scan. Based on these criteria, we included a total of 384 participants (Table 1). Additionally, we selected a cognitively unimpaired, amyloid-negative group who remained amyloid-negative and did not convert to mild cognitive impairment or dementia during follow-up (n = 111, Table 1). This group is used as a control group.

**Table 1:** Demographic and clinical characteristics.

	All AD	AD-Memory	AD-Executive	AD-Language	AD-Visuospatial	AD-No Domains	AD-Multiple	Control
N (% of all AD)	384	135 (35)	8 (2)	22 (6)	44 (11)	160 (42)	15 (4)	110
Age^a^	76.9 (5.9)	76.3 (6.0)	77.9 (7.3)	81.0 (6.1)	76.1 (5.9)	77.2 (5.7)	75.1 (4.2)	74.1 (6.5)
Sex, female (%)	168 (43.8)	61 (45.2)	2 (25.0)	7 (31.8)	14 (31.8)	77 (48.1)	7 (46.7)	54 (48.6)
*APOE*ε4, positive (%)	270 (70.5)	99 (73.9)	5 (62.5)	13 (59.1)	31 (70.5)	111 (69.4)	11 (73.3)	19 (17.3)
Incident AD (%)	213 (55.5)	80 (59.3)	3 (37.5)	12 (54.5)	27 (61.4)	83 (51.9)	8 (53.3)	–
Education, years	15.5 (2.9)	15.6 (2.8)	16.3 (3.3)	14.9 (2.8)	15.9 (2.7)	15.3 (3.1)	16.5 (2.3)	16.5 (2.8)
Left-handedness (%)	27 (7.0)	14 (10.4)	1 (12.5)	4 (18.2)	0 (0.0)	7 (4.4)	1 (6.7)	13 (11.7)
MMSE^b^	23.5 (2.9)	23.2 (2.7)	23.1 (2.2)	23.4 (3.5)	23.4 (3.5)	23.9 (2.9)	23.5 (2.3)	29.2 (1.1)
Whole brain FDG SUVR^c^	1.29 (0.13)	1.30 (0.11)	1.20 (0.14)	1.24 (0.14)	1.29 (0.12)	1.28 (0.15)	1.28 (0.14)	1.43 (0.13)

Values depicted are mean (SD), unless otherwise indicated. SUVR-standardized uptake value ratio. Pairwise differences between all groups are provided in Table A1.

a – Age at time of dementia diagnosis for AD-subgroups and age at first FDG scan for controls.

b – MMSE at time of dementia diagnosis for AD-subgroups and MMSE at first FDG scan for controls.

c – Whole brain FDG-SUVR for scan at time of dementia diagnosis (±12 months; n = 283) for AD-subgroups and at first FDG-scan for controls (n = 110).

### 2.2 Standard protocol approvals, registrations, and patient consents

Written informed consent was obtained for all participants, and study procedures were approved by the institutional review board at each of the participating centers. ADNI is listed in the ClinicalTrials.gov registry (ADNI-1: NCT00106899; ADNI-GO: NCT01078636; ADNI-2: NCT0123197).

### 2.3 Cognition

Detailed methods for obtaining cognitive scores and classifying ADNI participants with AD dementia into cognitively defined subgroups have been published (Crane et al., 2017; Mukherjee et al., 2020). Briefly, neuropsychological test data were obtained at time of AD dementia diagnosis. This could be either at inclusion into the ADNI cohort for people with prevalent AD or at any of the follow-up visits when an individual converted to dementia for people with MCI or (rarely) normal cognition. Individual elements from ADNI’s neuropsychological battery were categorized into one of four domains – memory, executive function, language or visuospatial function – by an expert panel (ET, JM, AS). For each domain, we used confirmatory factor analysis models in Mplus (Muthén and Muthén, 1998) to co-calibrate data from ADNI together with data from our legacy cohort (the Rush Memory and Aging Project [MAP] and Religious Orders Study [ROS] and the Adult Change in Thought [ACT] cohort) (Crane et al., 2017). We then transformed scores from ADNI to the metric obtained from ACT. The final transformed scores are scaled in SD units from ACT; a memory score of + 1 represents a score 1 SD above the mean memory score for people with incident AD in the ACT study, and an executive functioning score of −1 represents a score 1 SD below the mean executive functioning score for people with incident AD in the ACT study. ACT is a prospective cohort study that included 825 incident AD dementia cases at the time of analysis and was used as the reference because it was the largest prospective cohort of late-onset AD available to us (Crane et al., 2017). Further details on processing of neuropsychological data are provided in the supplement (Supplemental Text 1) and our previous publications and their supplementary materials (Crane et al., 2017, Mukherjee et al., 2020).

### 2.4 Subgroups

**Fig. A1:**
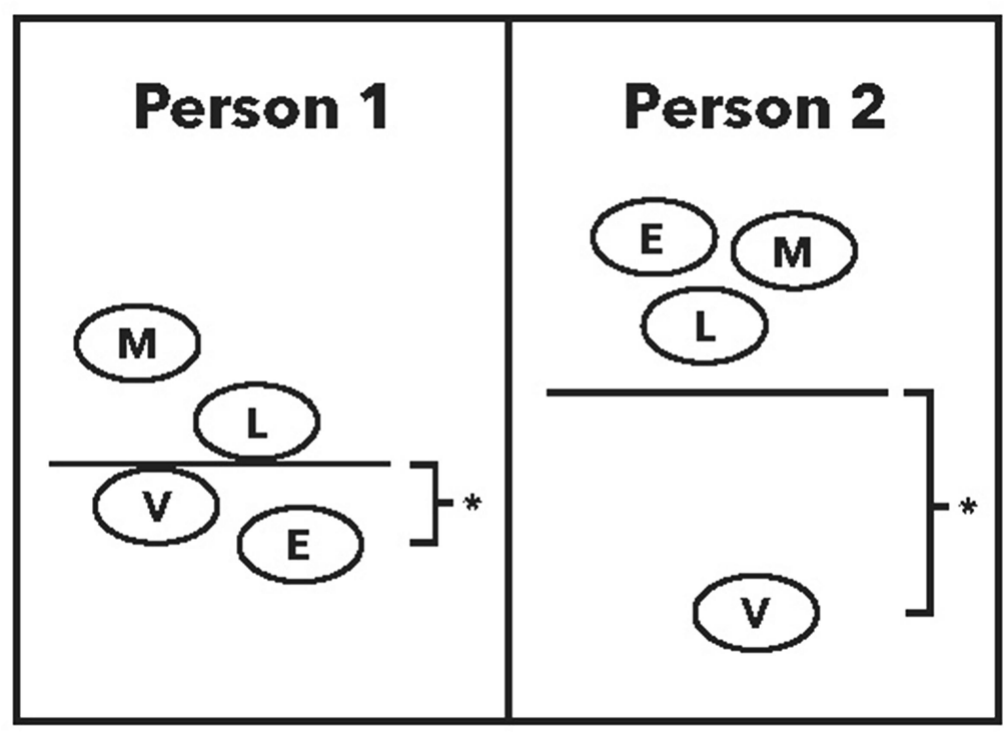
Graphical representation of how subgroup categorization was achieved. These ovals depict scores ranging from low (bottom) to high (top) for memory (M), executive functioning (E), language (L) and visuospatial functioning (V) at the time of Alzheimer’s disease dementia diagnosis. Person 1’s scores are clustered closely together. Person 2’s V score is much lower than their other scores. The average across domains is shown by the horizontal line. We consider differences from that average, shown by the brackets and asterisks, to place each person into a subgroup. Person 1: AD-No Domain. Person 2: AD-Visuospatial.

Classification into subgroups is based on the relative distribution of cognitive impairments across the four cognitive domains at the time of dementia diagnosis. First, we determine each participant’s individual average score across memory, executive functioning, language, and visuospatial functioning. We then determine deviations from that average for each domain. From a range of candidate thresholds, we previously determined a threshold of 0.8 SD to define substantial impairment in a given cognitive domain relative to the average across domains (Crane et al., 2017, Mukherjee et al., 2020). Classification is then achieved by determining which, and how many, domains(s) have relative impairments that exceed that threshold, yielding the following subgroups characterized by the index domain that showed relative impairment: isolated relative impairments in memory (AD-Memory), executive function (AD-Executive), language (AD-Language), or visuospatial function (AD-Visuospatial), relative impairments in multiple domains (>1 domain with relative impairment; AD-Multiple Domains), and no domains with relative impairments (AD-No Domains). A graphical representation of how subgroup classification was achieved is provided in Fig. A1. As classification is achieved by assessing relative intra-individual impairments, subgroup membership is determined by cognitive profiles rather than overall severity of impairments. For instance, membership in the AD-No Domains group does not indicate less overall impairment than membership in other subgroups, but rather that impairments in each domain are fairly similar to those of the other domains at the time of AD diagnosis. AD-Multiple Domains is a heterogeneous group with relative impairments across various and distinct domains. Therefore, we focus our analyses here on the other subgroups and show results for the AD-Multiple Domains group in Fig. A3.

### 2.5 Imaging analysis

**Fig. A2:**
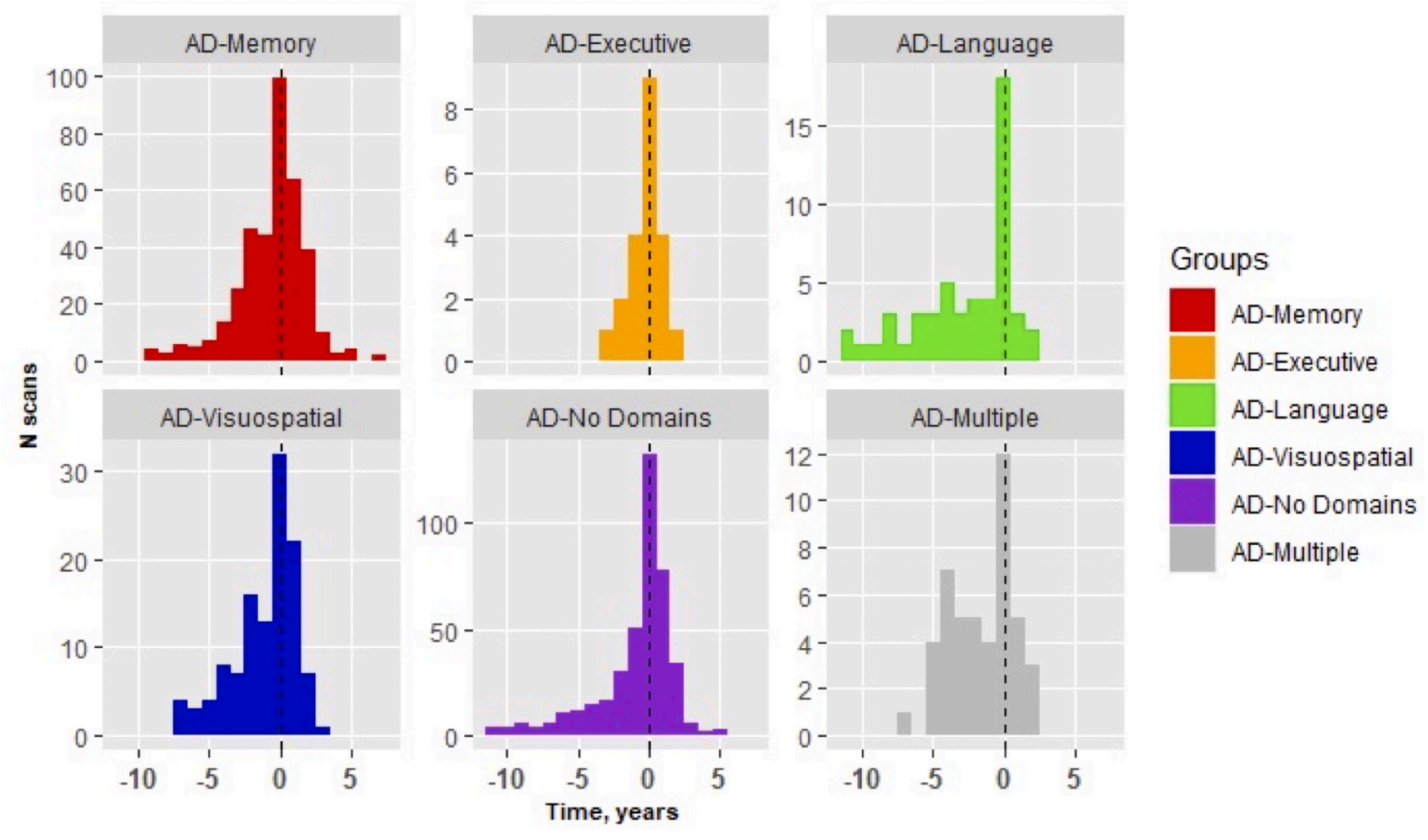
Number of scans available across time according to AD-subgroup. Time = 0 corresponds to time of AD dementia diagnosis. Note that the y-axis is on a different scale for each of the AD-subgroups.

**Fig. A3:**
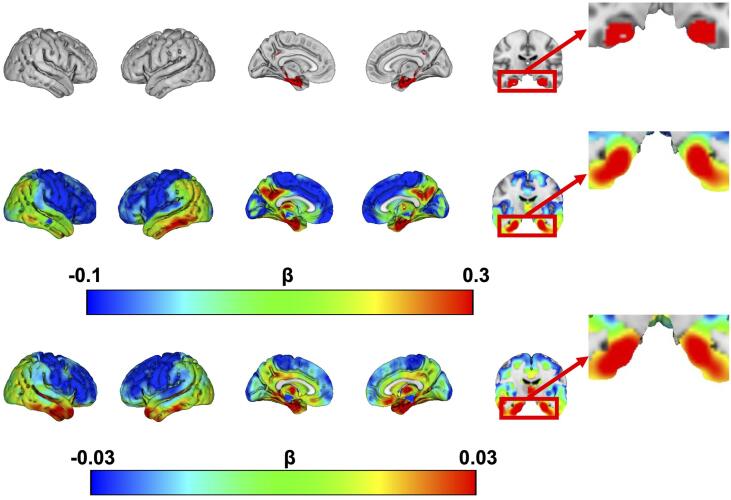
Voxel based results for AD-Multiple Domains. The top row represents significant differences in FDG signal between AD-Multiple Domains and cognitively normal controls, at p < 0.05 family-wise error corrected. The middle row represents the β coefficient from the same comparisons, this β value is not dependent on sample size. The bottom row represents the effect of time (β) on voxel wise FDG within the AD-Multiple Domains group.

[^18^F]FDG-PET images were acquired according to standardized scanning parameters (see: http://adni.loni.usc.edu/methods/pet-analysis-method/pet-analysis/ for details). [^18^F]FDG-PET images at time of dementia diagnosis (±12 months; mean time from diagnosis 0.20 ± 0.28 years) were used to assess hypometabolism patterns at time of diagnosis (t = 0) and were available for 293 participants (see section 2.6). In total, 1028 scans were available across all AD-participants and all of these were used to assess longitudinal trajectories of metabolism (see section 2.6). This 1028 includes the 293 scans at time of dementia diagnosis, 435 scans from before an AD diagnosis was established (mean time before diagnosis 3.17 ± 2.44 years) and 300 from after an AD diagnosis was established (mean time after diagnosis 1.46 ± 1.03 years). Fig. A2. And Table A2 give an overview of the number of scans available across subjects, groups and time. The mean total time between the first and last scans was 1.6 ± 1.8 years (range: 0–7 years) and mean time between two consecutive scans was 0.9 ± 0.7 years (range 0–4 years). For the controls, a total of 283 scans were available. For this group, the first available scan was used to determine t = 0. For the controls, one-hundred and ten scans were available at t = 0 (one for each control), which were used as a reference to determine hypometabolism at time of dementia diagnosis. For the controls, an additional 173 (mean follow-up 2.00 ± 1.40 years) scans were available after t = 0 and these were used, along with the t = 0 scans, to determine normative change over time in regional metabolism (see section 2.6).

We co-registered all [^18^F]FDG-PET images to structural T1-weighted MRI images obtained a maximum of ± 6 months from the acquisition date for each of the FDG-scans. Structural MRI was usually performed on the same day or within a few days of the FDG scan. Structural MRI images were acquired according to standardized protocols (see http://adni.loni.usc.edu/methods/mri-tool/mri-analysis/ for details). MRI images were first segmented into gray matter, white matter and CSF volumes. The gray matter images were then spatially normalized to MNI space using a standardized SPM12-based pipeline (Groot et al., 2018b). Normalization parameters from the MR images were then used to normalize corresponding [^18^F]FDG-PET images. To account for inter-individual differences in overall FDG-signal intensity, we converted the normalized FDG-scans into standardized uptake value ratio (SUVR) images using the mean tracer retention values in the pons (Nugent et al., 2020). The pons reference region-of-interest (ROI) was manually delineated on the MNI (MRI) template and mean tracer retention values within the pons for all FDG-scans were obtained after normalization to stereotactic MNI standard space. For ROI analyses, the Automated Anatomical Labelling (AAL) atlas was used to compute SUVRs in pre-specified ROIs that encompassed the gray matter of the main cerebral lobes: medial temporal lobe (MTL; hippocampus; amygdala; parahippcampal gyrus), frontal lobe (superior, middle and inferior frontal gyri; orbitofrontal and rectus gyri; frontal opercula; insular cortex; anterior and middle cingular gyri; para- and precentral lobules), temporal lobe (fusiform, Heschl’s-gyri; superior, middle and inferior temporal gyri; temporal pole), parietal lobe (superior and inferior parietal gyri; supramarginal and angular gyri) and occipital lobe (calcarine sulcus; cuneus; lingual; superior, middle and inferior occipital gyri) (Groot et al., 2018a). Due to their significance in the assessment of [^18^F]FDG-PET for dementia diagnosis (Rice and Bisdas, 2017) we examined the posterior cingulate and precuneus as two separate ROIs. Furthermore, given the association between language impairments and left-lateralized hypometabolism in lvPPA (Lehmann et al., 2013) and subtle right-lateralization of atrophy in PCA (Groot et al., 2020), we also computed an asymmetry index for an AD-specific ROI (temporoparietal [TPC] (Ossenkoppele et al., 2015a) combining the parietal, precuneus and temporal ROIs enumerated above) to evaluate lateralization of hypometabolism in each AD-subgroup. This TPC asymmetry index was calculated as ([right TPC – left TPC]/[right TPC + left TPC]), higher values indicate more metabolism in the right-hemisphere compared to the left and negative values mean the opposite.

Differences in overall degree of hypometabolism between groups might drive differences in hypometabolism patterns between AD-subgroups. Furthermore, clinical assessment of FDG-scans primarily relies on relative regional hypometabolism rather than overall levels of metabolism. Therefore, in order to account for overall levels of metabolism, we combined all cerebral AAL atlas regions into one combined global ROI and added this global FDG measure as a covariate in the statistical analyses (see section 2.6).

### 2.6 Statistical analyses

Statistical analyses were performed in SPM12 and R version 3.5.2. To visualize overall spatial patterns of hypometabolism at time of dementia diagnosis for each subgroup we assessed voxelwise general linear models comparing FDG scans at time of diagnosis against the control group, adjusting for age, sex and whole brain FDG-SUVR, as well as the time-lag between date of diagnosis and date of FDG-scan. We applied a significance threshold of p = 0.05 with family-wise error correction to produce binarized, voxelwise maps highlighting voxels with significant hypometabolism compared to controls. As differences in group size between the subgroups affect the power to detect significant voxels in these comparisons, we also present voxelwise β-coefficient maps (representing the group effect in the models) that are not affected by differences in sample size across groups. Because these models were adjusted for whole-brain FDG-SUVR, the β coefficient for group difference between AD-subgroups and controls indicates differences in metabolism relative to global levels of metabolism.

Longitudinal trajectories of hypometabolism across subgroups were visualized by voxelwise linear mixed model analyses performed in SPM12. These models were adjusted for age, sex, time (from dementia diagnosis) and whole brain gray matter FDG-SUVR. To highlight the change over time, we visualize the β-coefficient of time in each gray matter voxel across the brain. Because these models were adjusted for whole-brain FDG-SUVR, the β coefficient for time indicates change in metabolism over time relative to global levels of metabolism change.

To formally test for differences in longitudinal trajectories of hypometabolism within the 6 ROIs between the AD-subgroups, we fit linear mixed-effect models with random intercept and slopes for individuals using the “lme4” package in R. We included one term for all subgroups to predict regional FDG-SUVR, and pairwise differences between groups were assessed by the time*group interaction effect. These models were also adjusted for age, sex, time and whole brain gray matter [^18^F]FDG-PET-SUVR.

In an additional analysis we assessed whether regional differences in (change in) metabolism between subgroups were influenced by whether cases were prevalent AD dementia (diagnosed at first study visit) or incident AD dementia (diagnosed at any of the follow-up visits). To examine this, we ran the same models as before but added an additional interaction term group*[incident/prevalent AD] and a three-way interaction term time*group*[incident/prevalent AD]. Effects of prevalent and incident AD dementia are visualized by running the initial models while stratifying for prevalent vs incident AD.

**Fig. A4:**
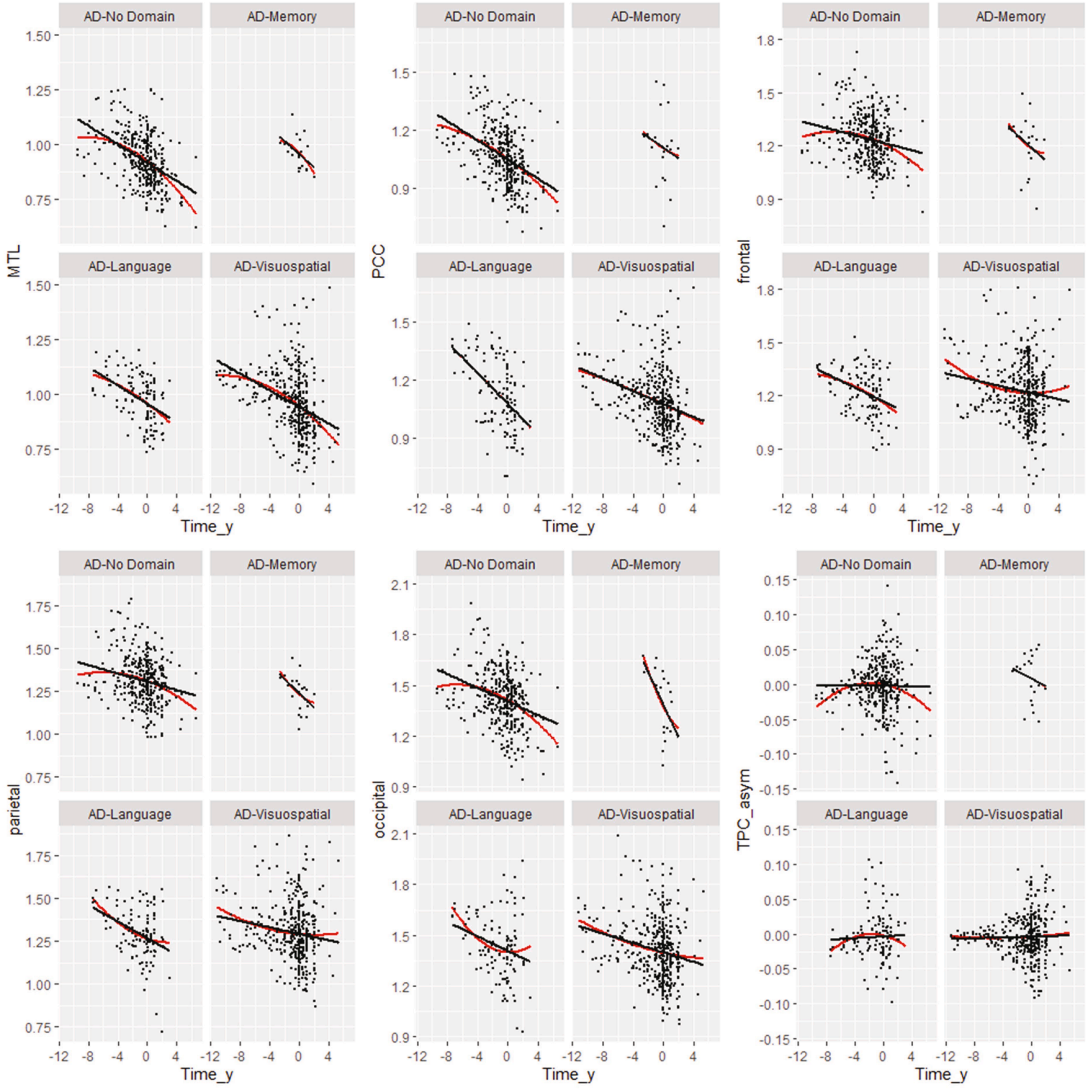
Distribution of FDG signal within regions-of-interest across the AD-subgroups. The black line indicates the regression slope from an univariate linear regression and the red line indicates the slope from an univariate 2^nd^-degree polynomial model. Slopes for 3^rd^-degree polynomial models were very similar to the slopes in the 2^nd^-degree polynomial models and are not shown in the figure.

Because the AD-Executive group only had 6 participants with longitudinal FDG measurements, this group was not included in the ROI-based linear mixed model analyses. We present voxelwise analyses from this group but results should be interpreted with caution due to the small sample size. We also tested non-linear (2nd-degree polynomial) models (Fig. A4) but the data showed that linear models were the best fit to our data; we therefore present linear model-based results here.

## 3 Results

Of the 384 participants, 135 (35%) were categorized as AD-Memory, 8 (2%) as AD-Executive, 22 (6%) as AD-Language, 44 (11%) as AD-Visuospatial, 15 (4%) as AD-Multiple Domains, and 160 (42%) as AD-No Domains. Demographic and clinical characteristics of the sample at time of AD dementia diagnosis are displayed in Table 1. We provide all pairwise comparisons between AD-subgroups and between AD-subgroups and controls in Table A1. Age was, on average, higher in the AD-Language subgroup compared to AD-Memory, AD-Visuospatial and AD-No Domains. There were no differences between the AD-subgroups on any of the other characteristics. Controls were, on average, younger than AD-Memory, AD-Language and AD-No Domains. Furthermore, controls were also less often *APOE*ε4 positive

### 3.1 Voxelwise differences in hypometabolism at time of AD diagnosis

**Fig. 1:**
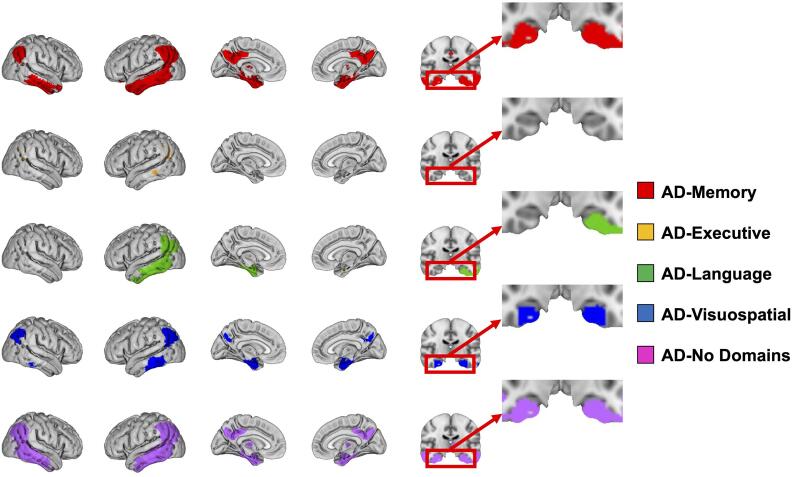
Significant differences in metabolism between AD-subgroups and controls at time of AD dementia diagnosis. Threshold is at p < 0.05, family-wise error corrected and adjusted for age, sex, whole brain FDG-SUVR and time-lag between diagnosis and scan.

**Fig. 2:**
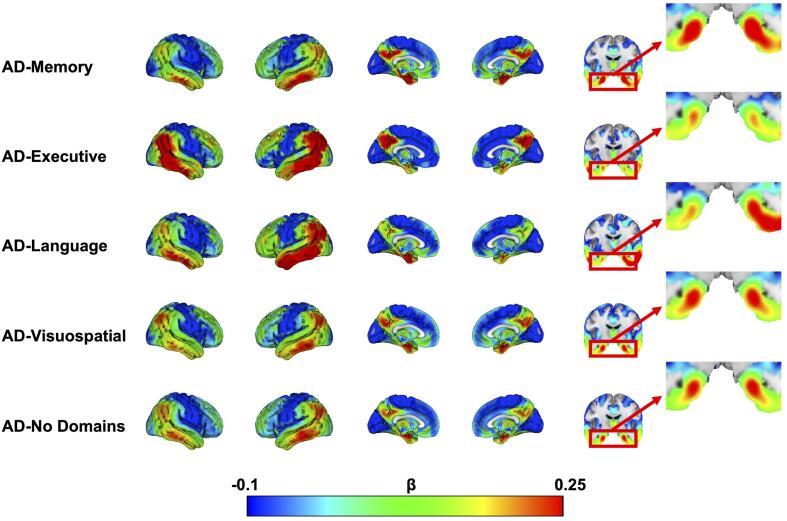
β-maps indicating the strength of the difference between AD-subgroups and controls. As the binary significance maps depicted in Fig. 1 are heavily dependent on sample size differences between AD-subgroups, we additionally display the β-maps from the same comparisons, representing the strength of the association, which are not dependent on sample size.

Voxelwise contrasts between controls and AD-subgroups at time of dementia diagnosis revealed a temporoparietal pattern with involvement of the posterior cingulate across all subgroups, a pattern which is typically observed in AD (Fig. 1). Furthermore, we observed subgroup specific regions of hypometabolism. Specifically, we visually observed more medial temporal lobe (MTL) involvement in AD-Memory, marked asymmetry (left > right hypometabolism) in AD-Language, more hypometabolism in the frontal area for AD-Executive and somewhat more prominent parietal than temporal hypometabolism in AD-Visuospatial (Fig. 1, Fig. 2).

### 3.2 Voxelwise differences in longitudinal change in metabolism

**Fig. 3:**
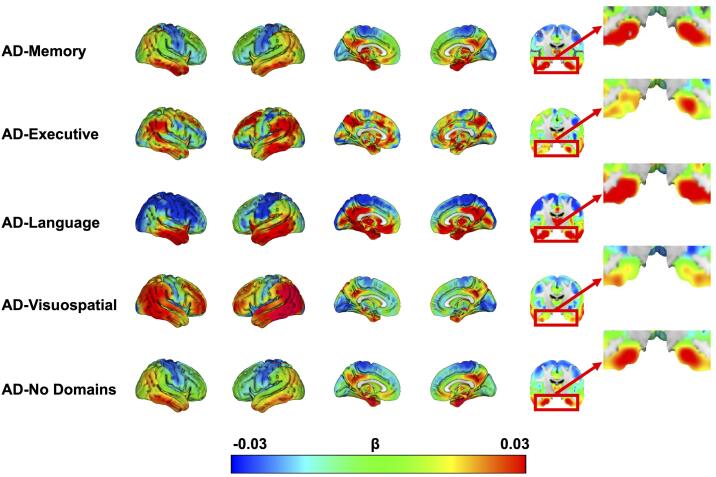
Longitudinal change in glucose hypometabolism across AD-subgroups. The β coefficient is the effect of time from linear mixed model analyses predicting voxel-wise FDG, corrected for age, sex and whole-brain FDG-SUVR. Because these models were adjusted for whole-brain FDG-SUVR, the β coefficients indicate change in metabolism over time relative to the overall levels of metabolism. We did not apply any threshold when visualizing these effects, rather the whole spectrum of the association is shown.

The effects of time from the voxelwise linear mixed model analyses, indicating longitudinal change in brain metabolism across subgroups, again revealed a temporo-parietal pattern with posterior cingulate involvement for all AD-subgroups (Fig. 3). This indicates that, in accordance with the hypometabolism observed at dementia diagnosis, decline in metabolism is fastest in these regions. Visual inspection of the β-maps (voxelwise β-coefficient for time) also reveals regions that demonstrated relatively faster decline in metabolism in one subgroup compared to the others. Specifically, we observed faster bilateral decline of MTL metabolism in AD-Memory compared to AD-Executive and AD-Visuospatial. MTL decline in metabolism was more pronounced in the right-hemisphere in AD-Memory compared to AD-Language but left-MTL metabolism decline was comparable between these two groups. Furthermore, temporal decline in AD-Language was pronounced and asymmetrical (faster decline on the left). The AD-Visuospatial subgroup showed a widespread cortical pattern of decline in metabolism, which included frontal and posterior regions that were more affected than in the other groups (Fig. 3).

#### 3.2.1 Region-of-interest analyses

**Fig. 4:**
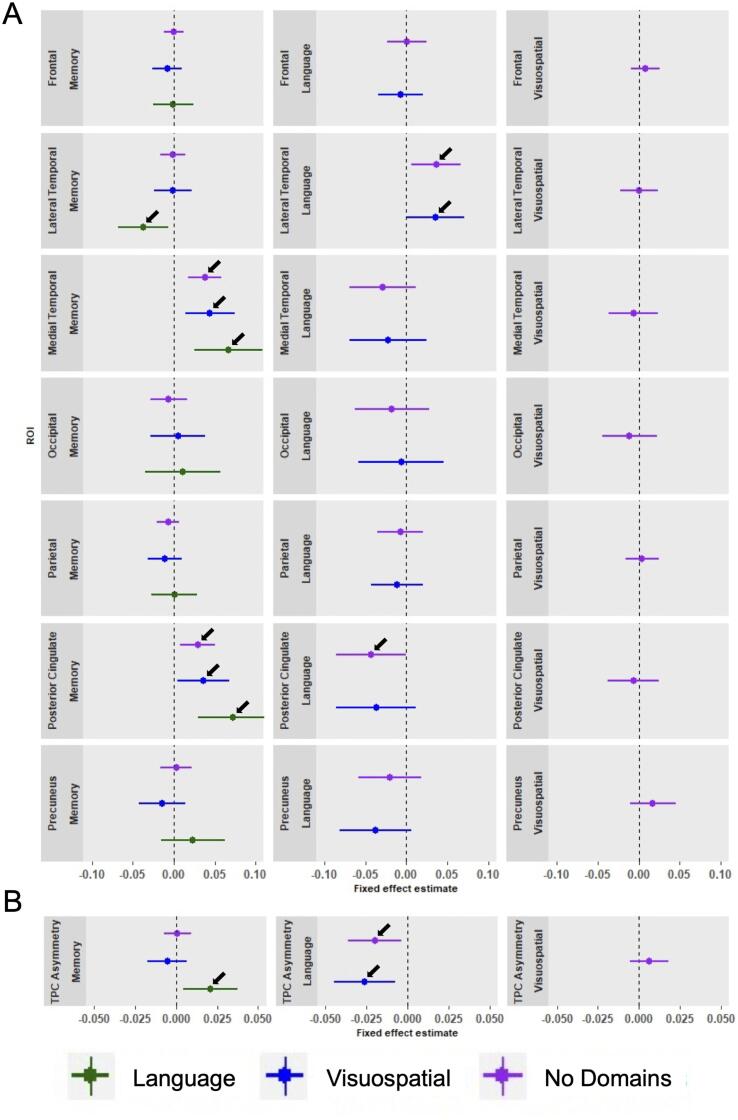
Differences in metabolism within composite regions of interest across subgroups at time of AD diagnosis. The effects displayed are the fixed group effects from our linear mixed effects models using the AD-subgroup displayed on the left for reference to assess the difference with the group indicated with colors. These group effect beta-coefficients indicate the overall difference in regional FDG between subgroups. Models were adjusted for age and sex effects, as well as for whole brain FDG SUVR to adjust for possible differences in global hypometabolism between subgroups. The 95% CI not overlapping with zero indicates a significant effect. A positive effect in panel A indicates that the colored group has greater brain metabolism than the reference, and a negative effect the opposite. For instance, in the left-most medial temporal cortex panel, the blue effect means that AD-Visuospatial has more metabolism in that ROI than AD-Memory. Positive effects in panel B indicate that the colored group has a more left < right metabolic pattern than the reference, and negative means the opposite. For instance, in the left panel the negative green effect signifies that AD-Language has a more left < right TPC brain metabolism pattern than AD-Memory. A voxelwise representation of the AD-subgroup differences at baseline, using the AD-No Domains group as the references, is provided in Fig. A5, Fig. A6.

Linear mixed effects analyses assessing brain metabolism within ROIs revealed differences in regional hypometabolism at time of AD diagnosis and differences in rates of decline in regional metabolism between subgroups. Specifically, we observed that medial temporal and posterior cingulate metabolism was lower in AD-Memory compared to all other groups. Furthermore, lateral temporal metabolism was lower in AD-Language compared to all other groups and the TPC asymmetry index reveal that AD-Language is characterized by a more asymmetrical (left < right metabolism) pattern than all other subgroups (Fig. 4A; Fig. 4B; Fig. 6).

**Fig. 5:**
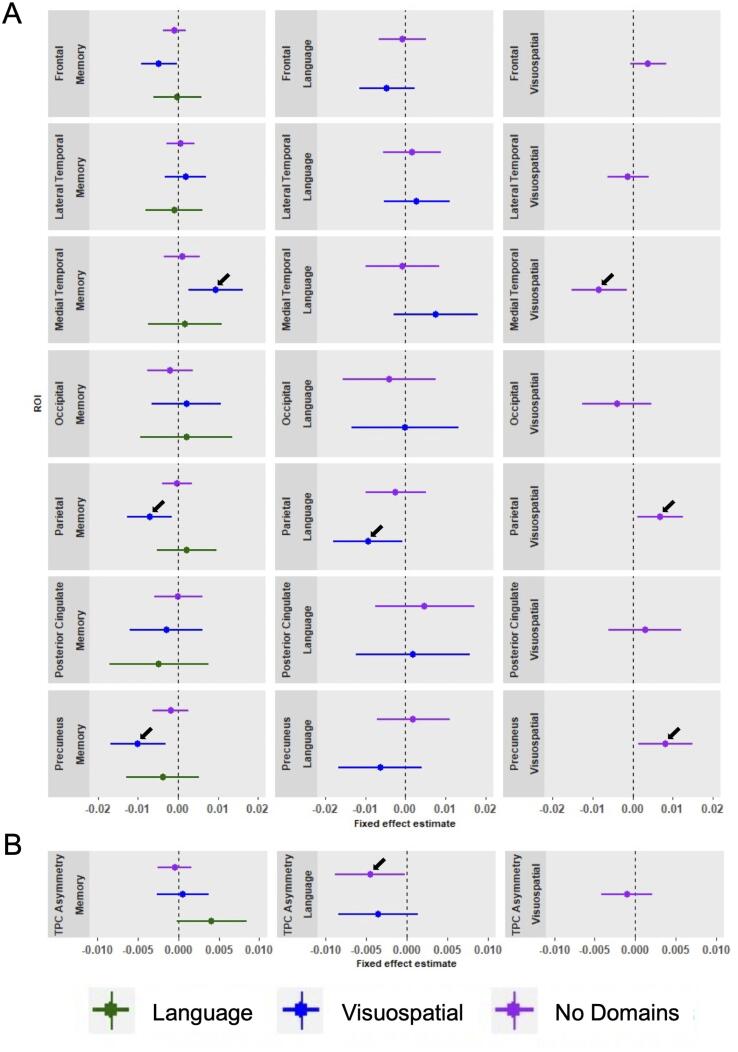
Differences in decline in metabolism over time within composite regions of interest across subgroups. The effects displayed are the group*time interaction effects from linear mixed effects models using the AD-subgroup displayed on the left for reference to assess the difference with the colored group. These group*time effect beta-coefficients indicate differences in change over time in FDG across all available timepoints. All models were adjusted for age and sex effects, as well as for whole brain FDG SUVR to adjust for possible differences in global hypometabolism between subgroups. The 95% CI not touching x = 0 indicates a significant effect. A positive effect indicates that the colored group has slower decline in brain metabolism than the reference, and a negative effect a faster decline in brain metabolism than the reference. For instance, in the left-most medial temporal panel, the blue effect means that AD-Visuospatial has slower decline in brain metabolism in that ROI than AD-Memory. Positive effects in panel B indicate that the TPC pattern in the colored group becomes more asymmetrical (left < right) over time compared to the reference, and negative effects mean the opposite. For instance, the purple effect in the middle panel indicates that TPC metabolism in AD-No Domains becomes more asymmetrical over time (left > right) with time compared to AD-Language. A voxelwise representation of the AD-subgroup differences in terms of change in metabolism over time, using the AD-No Domains group as the references, is provided in Fig. A7.

**Fig. 6:**
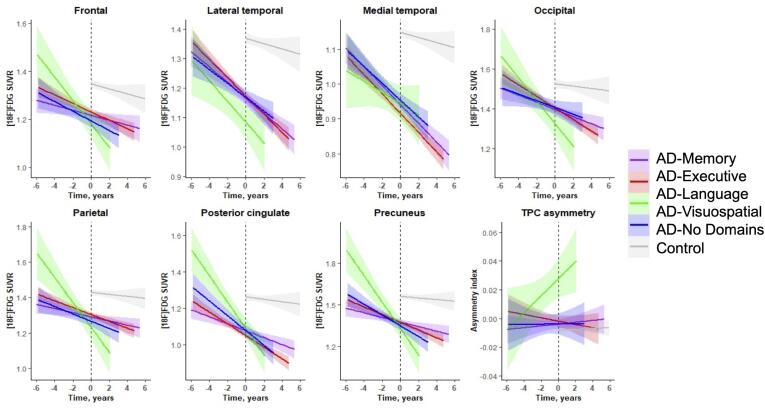
Longitudinal hypometabolism within regions-of-interest across subgroups.

Fig. 5A and B display differences in rates of decline in metabolism between AD-subgroups. We found that MTL metabolism declined faster in AD-Memory compared to AD-Visuospatial and AD-No Domains. Parietal decline in metabolism was faster in AD-Visuospatial compared to all other groups and metabolism decline in the precuneus was faster in AD-Visuospatial compared to AD-Memory and AD-No Domains (Fig. 5A).

Differences in change in TPC asymmetry over time indicated that metabolism in the TPC became more left-lateralized as time progressed in AD-Language compared to AD-No Domains (Fig. 5B; Fig. 6).

#### 3.2.2 Incident vs prevalent AD cases

**Fig. A8:**
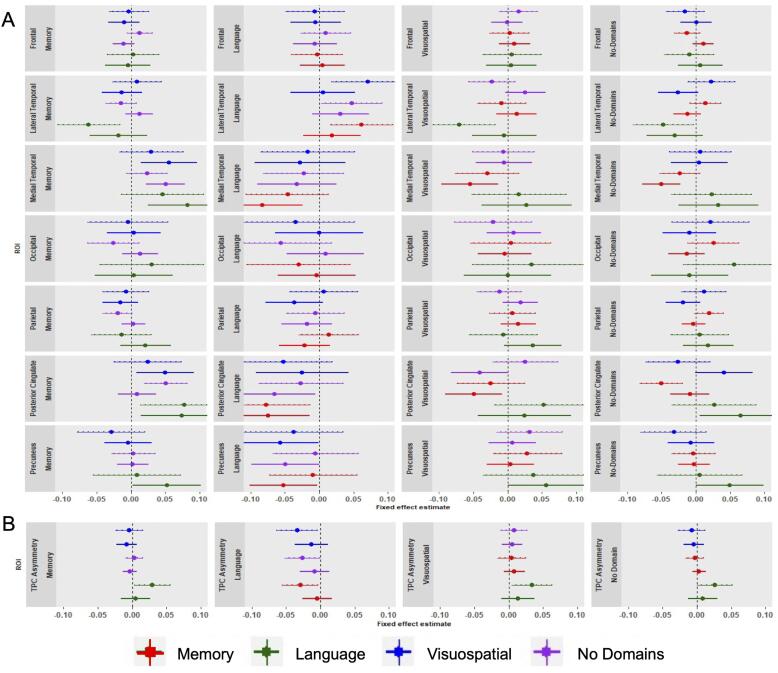
Differences in metabolism within composite regions of interest across subgroups at time of AD diagnosis, stratified for incident vs prevalent AD dementia cases. The effects displayed are the fixed group effects from our linear mixed effects models using the AD-subgroup displayed on the left for reference to assess the difference with the group indicated with colors. Incident AD dementia cases are displayed in solid bars and prevalent AD dementia cases with dotted bars. These group effect β-coefficients indicate the overall difference in regional FDG between subgroups. Models were adjusted for age and sex effects, as well as for whole brain FDG SUVR to adjust for possible differences in global hypometabolism between subgroups. The 95% CI not overlapping with zero indicates a significant effect. A positive effect in panel A indicates that the colored group has greater brain metabolism than the reference, and a negative effect the opposite. For instance, in the left-most lateral temporal panel, the dotted green effect means that prevalent cases in the AD-Language group has lower metabolism in that ROI than prevalent cases in the AD-Memory group. Positive effects in panel B indicate that the colored group has a more left<right metabolic pattern than the reference, and negative effects mean the opposite. For instance, in the left panel the dotted green effect signifies that prevalent cases in the AD-Language group have a more left<right TPC brain metabolism pattern than prevalent cases in the AD-Memory group.

**Fig. A9:**
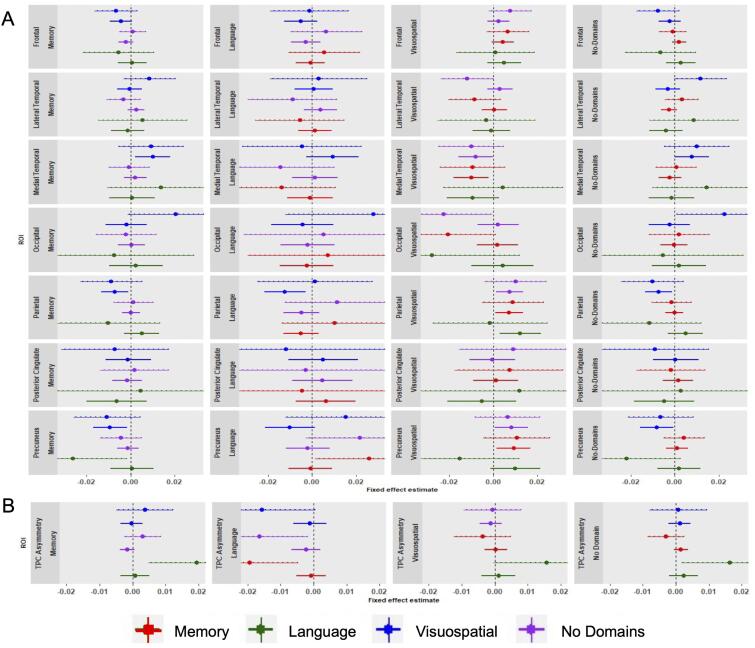
Differences in decline in metabolism over time within composite regions of interest across subgroups, stratified for incident vs prevalent AD dementia cases. The effects displayed are the group*time interaction effects from linear mixed effects models using the AD-subgroup displayed on the left for reference to assess the difference with the colored group. Incident AD dementia cases are displayed in solid bars and prevalent AD dementia cases with dotted bars. These group*time effect β-coefficients indicate differences in change over time in FDG across all available timepoints. All models were adjusted for age and sex effects, as well as for whole brain FDG SUVR to adjust for possible differences in global hypometabolism between subgroups. The 95% CI not touching x=0 indicates a significant effect. A positive effect indicates that the colored group has slower decline in brain metabolism than the reference, and a negative effect a faster decline in brain metabolism than the reference. For instance, in the left-most precuneus panel, the dotted green effect means that prevalent cases in the AD-Language group have faster decline in brain metabolism in this ROI than AD-Memory. Positive effects in panel B indicate that the TPC pattern in the colored group becomes more asymmetrical (left<right) over time compared to the reference. For instance, the dotted green effect in the left panel indicates that TPC metabolism in prevalent cases in the AD-Language group show a metabolism pattern that becomes more asymmetrical over time (left<right) compared to AD-Memory.

Incident vs prevalent AD dementia had a significant effect on differences in occipital metabolism decline between AD-Visuospatial and AD-No Domains (0.070, p = 0.03), such that faster decline in AD-Visuospatial was only observed in prevalent AD dementia but not in incident AD dementia (Fig. A8A). Furthermore, there was an interaction effect of [incident/prevalent AD dementia]*time*group on TPC asymmetry of metabolism, which indicated that TPC asymmetry (left < right metabolism) increased in AD-Language compared to AD-Memory (-0.019, p < 0.01), and AD-No Domains (-0.014, p = 0.04) in prevalent AD dementia but not in incident AD dementia (Fig. A9B).

Plotted slopes indicate change over time in raw [^18^F]FDG-PET-SUVRs and TPC asymmetry values (bottom right), with their 95% confidence interval. T = 0 corresponds to the time of dementia diagnosis.

## 4 Discussion

We used an established framework to categorize amyloid-β positive individuals with AD dementia into cognitive subgroups. We found that these subgroups displayed distinct patterns and trajectories of hypometabolism. Specifically, in AD-Memory we observed relatively pronounced MTL and posterior cingulate hypometabolism compared to all other groups. Furthermore, MTL decline in metabolism was faster in AD-Memory compared to AD-Visuospatial and AD-No Domains. In AD-Language, lateral temporal hypometabolism was worse and more asymmetrical (left < right metabolism) compared all other groups, and left-lateralization of hypometabolism became more pronounced with time compared to AD-No Domains. In AD-Visuospatial, parietal decline in metabolism was faster compared to all other subgroups and decline in precuneus metabolism was faster compared to AD-Memory and AD-No Domains. These observations regarding differences in hypometabolism patterns and patterns of longitudinal decline in metabolism within the spectrum of typical AD indicate that cognitive subgrouping yields biologically distinct groups.

### 4.1 Interpretation of results

Previous studies that implemented the same framework to categorize individuals have shown that there are clinical implications to subgroup membership, such as fewer depressive symptoms (Bauman et al., 2019), and slower functional and cognitive decline (Mez et al., 2013b, Mez et al., 2013a) in the AD-Memory group compared to the other subgroups. Furthermore, another investigation revealed that associations for known genetic risk factors for AD are different between the subgroups (Crane et al., 2017) and revealed 33 novel loci that were specifically associated to individual subgroups (Mukherjee et al., 2020), suggesting that genetic factors might be involved in the emergence of clinical differences between subgroups. With the present study, we extend on these findings by showing that these subgroups also show different patterns of brain metabolism and hypometabolism trajectories, as measured with [^18^F]FDG-PET.

[^18^F]FDG-PET has long been used as a diagnostic measure to detect AD and the AD-signature FDG pattern is characterized by temporoparietal, and posterior cingulate hypometabolism. In the present study, we confirmed this common pattern across all AD-subgroups. However, the subgroup-specific patterns indicate that there is clinical-neuroanatomical heterogeneity across the spectrum of typical late onset AD that can be detected by [^18^F]FDG-PET. The relatively greater medial temporal hypometabolism found in the AD-Memory group suggests that, in accordance to findings on MRI (Scheltens et al., 2017), relatively more medial temporal lobe involvement is associated with a phenotype with relatively more amnestic impairments. Furthermore, the relatively more pronounced posterior cingulate hypometabolism in AD-Memory indicates that posterior cingulate hypometabolism, which is characteristic of an AD-like FDG pattern (Laforce et al., 2018) and regarded as an early feature (Minoshima et al., 1997), might be more strongly related to an amnestic phenotype of AD than to non-amnestic phenotypes.

For AD-Language, we show that asymmetrical lateral temporal hypometabolism is prominent, which is in line with the hypometabolism pattern that is observed in lvPPA (Lehmann et al., 2013). A previous study has related an lvPPA AD-phentype to neurodevelopmental learning disabilities (i.e., dyslexia) (Miller et al., 2013), which is, in turn related to brain asymmetry (Geschwind and Galaburda, 1985). Whether premorbid dyslexia might partly underlie AD-Language subgroup membership in our sample is an intriguing possibility. Longer lead times of longitudinal FDG-PET data will be needed to determine whether left lateralization of hypometabolism among people who ultimately go on to develop AD-Language precedes accumulation of AD-pathology or whether the left-hemisphere is more vulnerable to hypometabolism decline after pathology has set in. For AD-Visuospatial we observed relatively pronounced hypometabolism in the lateral parietal lobe and precuneus. This radiological phenotype is in line to what is found in PCA (Crutch et al., 2017) and, analogous to dyslexia and lvPPA, a link between learning disabilities (e.g., dyscalculia) and PCA has been demonstrated (Miller et al., 2018). Taken together, pre-morbid differences in metabolism may play a role in determining subgroup membership, but this remains to be determined in future studies.

There was congruency of regional hypometabolism with the expected cognitive profiles that define each AD subgroup. Progressive medial temporal and posterior cingulate hypometabolism was notable in the AD-Memory subgroup. Progressive frontal lobe hypometabolism was visually observed in the AD-Executive group. Progressive asymmetric left-temporal hypometabolism was notable in the AD-Language subgroup. Progressive parietal and precuneus hypometabolism was notable in the AD-Visuospatial group. This indicates that, at least in theory, FDG could differentiate subgroups, which might inform future investigations that provide a deeper understanding of the mechanisms underlying the emergence of clinical heterogeneity in AD. Furthermore, when combined with biomarkers of AD-pathology (e.g., amyloid biomarkers), our findings regarding differential FDG-PET patterns and trajectories across subgroups could also improve diagnostic procedures, especially for individuals with a non-amnestic phenotype.

### 4.2 Strengths and limitations

Among the strengths of the present study are the relatively large sample of individuals from ADNI with FDG-PET available (N = 384), the assessment of longitudinal data, and the implementation of a classification scheme that categorizes people into theory-driven subgroups. The relatively simple method of classification we have used has the advantage of not relying on large samples to produce clusters of factor scores, and can be implemented on an individual basis (Crane et al., 2017). The present study also has several limitations. First, mean time between first and last scan across subjects was only 1.6 ± 1.8 years, and longer lead and follow-up times are likely needed to disentangle more subgroup-differences in longitudinal trajectories in metabolism. Furthermore, we were unable to formally assess decline in metabolism over time in the AD-Executive subgroup, because this group consisted of only six individuals with longitudinal data. The low prevalence of this subgroup among our sample is in accordance with the rarity of the dysexecutive variant of AD (Dickerson and Wolk, 2011, Ossenkoppele et al., 2015b, Townley et al., 2020). Similarly, we were unable to properly assess the cognitive vs neurobiological associations in the heterogeneous AD-Multiple Domains subgroup, as this relatively small group includes individuals with a range of different cognitive phenotypes. Also, due to the focus of ADNI on amnestic MCI and AD presentations, the relative prevalence of AD-subgroups in the present study may not be representative of other cohorts. Indeed, we published the proportions of people with late-onset AD in each subgroup in our prior paper (Mukherjee et al., 2020); ADNI had a higher proportion of people in the AD-Memory group than was seen in other studies. Follow-up examinations are needed in more diverse samples. Another potential limitation is we did not correct for partial volume effects, which might disproportionally affect regions with more atrophy. Furthermore, we were unable to formally assess whether differences in symptom duration at time of AD dementia diagnosis may have partly explained our results as there was no objective measure of symptom duration available. Our regional FDG results in stratified groups of prevalent and incident AD dementia showed that prevalent AD dementia may show more subgroup-differences (e.g., more TPC asymmetry in AD-Language) indicating that symptom duration might affect subgroup-specific metabolism and highlighting the need for further examination. Finally, the framework that was used to categorize individuals into subgroups is based exclusively on cognitive data and ignores behavioral and personality features, which are increasingly recognized as being part of the clinical presentation of AD.

## 5 Conclusions

We found differences in hypometabolism patterns and trajectories across groups of people in different cognitively-defined subgroups. This finding provides further support that there are biological differences between cognitively-defined subgroups. Further research is needed to determine whether differences in regional metabolism patterns we observed reflect differences in regional tau distribution (Murray et al., 2011, Whitwell et al., 2008) and/or amyloid-β (Lehmann et al., 2013) pathology. These developments may advance our growing knowledge on the fundamental mechanisms involved in the etiology of AD and the emergence of clinical and neurobiological heterogeneity among people with AD, and add to a growing literature documenting biological distinctions across cognitively-defined AD subgroups.

## Funding

This work was supported by R01 AG 029672 (Paul K Crane, PI). Wiesje van der Flier is recipient of JPND-funded E-DADS (ZonMW project #733051106). Frederik Barkhof is supported by the NIHR biomedical research center at UCLH. Jesse Mez is supported by P30AG13846 and K23AG046377. Research of Alzheimer center Amsterdam is part of the neurodegeneration research program of Amsterdam Neuroscience. Alzheimer Center Amsterdam is supported by Stichting Alzheimer Nederland and Stichting VUmc fonds. Wiesje van der Flier holds the Pasman chair. The clinical database structure was developed with funding from Stichting Dioraphte. The sponsors had no role in the writing of the report and in the decision to submit the article for publication.

## CRediT authorship contribution statement

**Colin Groot:** Conceptualization, Formal analysis, Investigation, Writing - original draft, Visualization. **Shannon L. Risacher:** Conceptualization, Methodology, Writing - review & editing. **J.Q. Alida Chen:** Data curation, Writing - review & editing. **Ellen Dicks:** Data curation, Writing - review & editing. **Andrew J. Saykin:** Writing - review & editing. **Christine L. Mac Donald:** Writing - review & editing. **Jesse Mez:** Writing - review & editing. **Emily H. Trittschuh:** Writing - review & editing. **Shubhabrata Mukherjee:** Data curation, formal analysis, Writing – review & editing. **Frederik Barkhof:** Writing - review & editing, Data curation. **Philip Scheltens:** Writing - review & editing, Data curation. **Wiesje M. van der Flier:** Writing - review & editing, Data curation, Supervision. **Rik Ossenkoppele:** Conceptualization, Methodology, Resources, Writing - original draft, Writing - review & editing, Supervision, Funding acquisition. **Paul K. Crane:** Conceptualization, Methodology, Resources, Writing - original draft, Writing - review & editing, Supervision, Funding acquisition.

## Declaration of Competing Interest

The authors declare that they have no known competing financial interests or personal relationships that could have appeared to influence the work reported in this paper.

## Figures and Tables

**Fig. A5:**
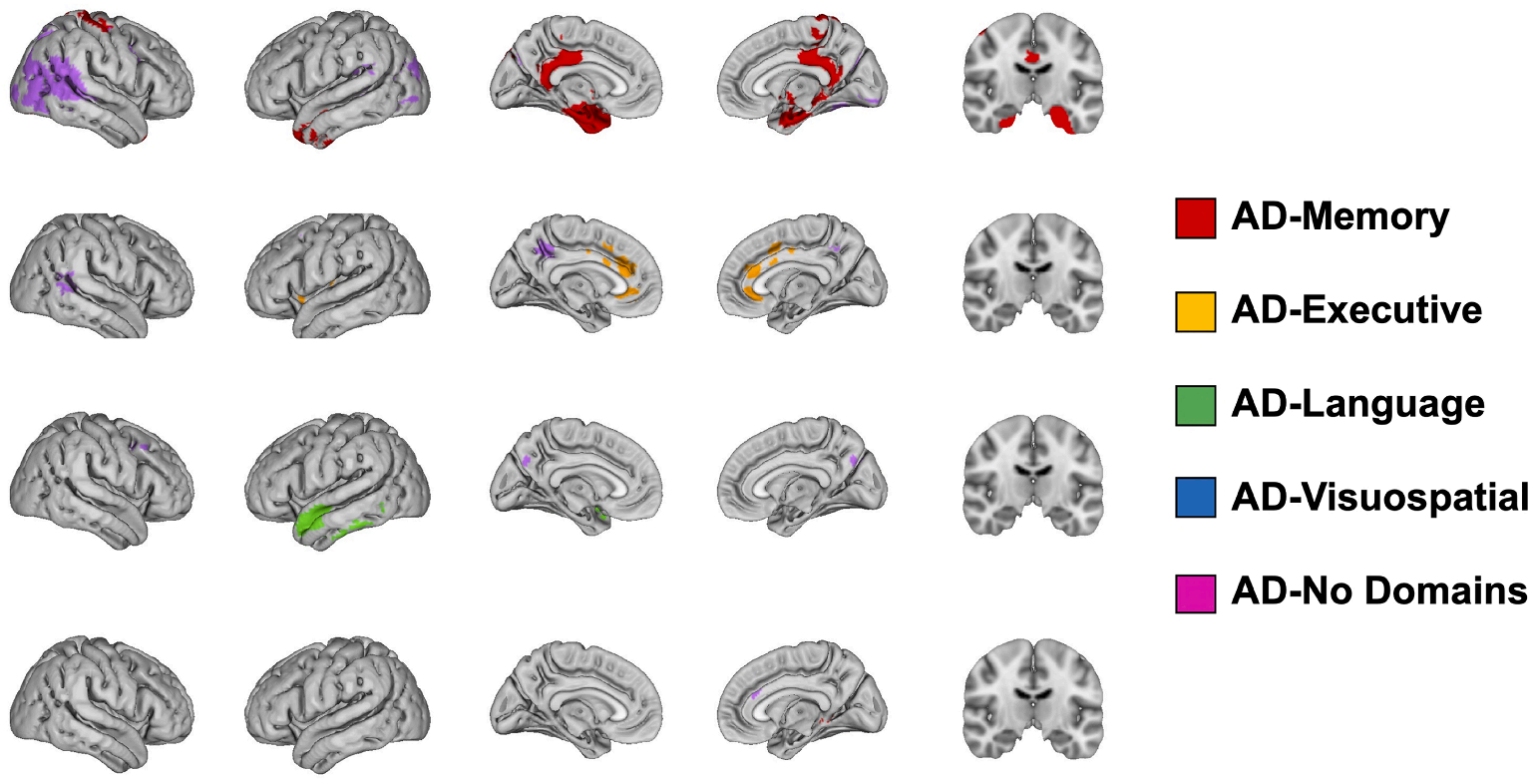
Significant differences in metabolism between AD-No Domains and the other AD-subgroups. Significance was set at p<0.01. Red, orange, green and blue colors indicate that the AD-subgroup had lower metabolism compared to AD-No domains and purple indicates that AD-No Domains had lower metabolism compared to the AD-subgroup.

**Fig. A6:**
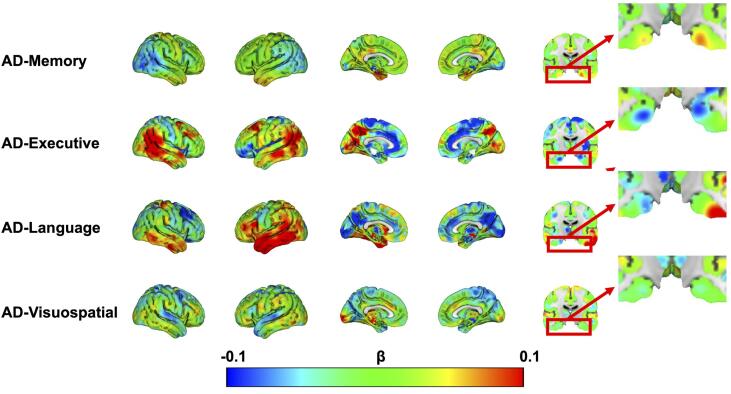
β-maps indicating the strength of the difference between AD-No domains and the other AD-subgroups. As the binary significance maps depicted in Fig. A5 are heavily dependent on sample size differences between AD-subgroups, we additionally display the β-maps from the same comparisons, representing the strength of the association, which are not dependent on sample size.

**Fig. A7:**
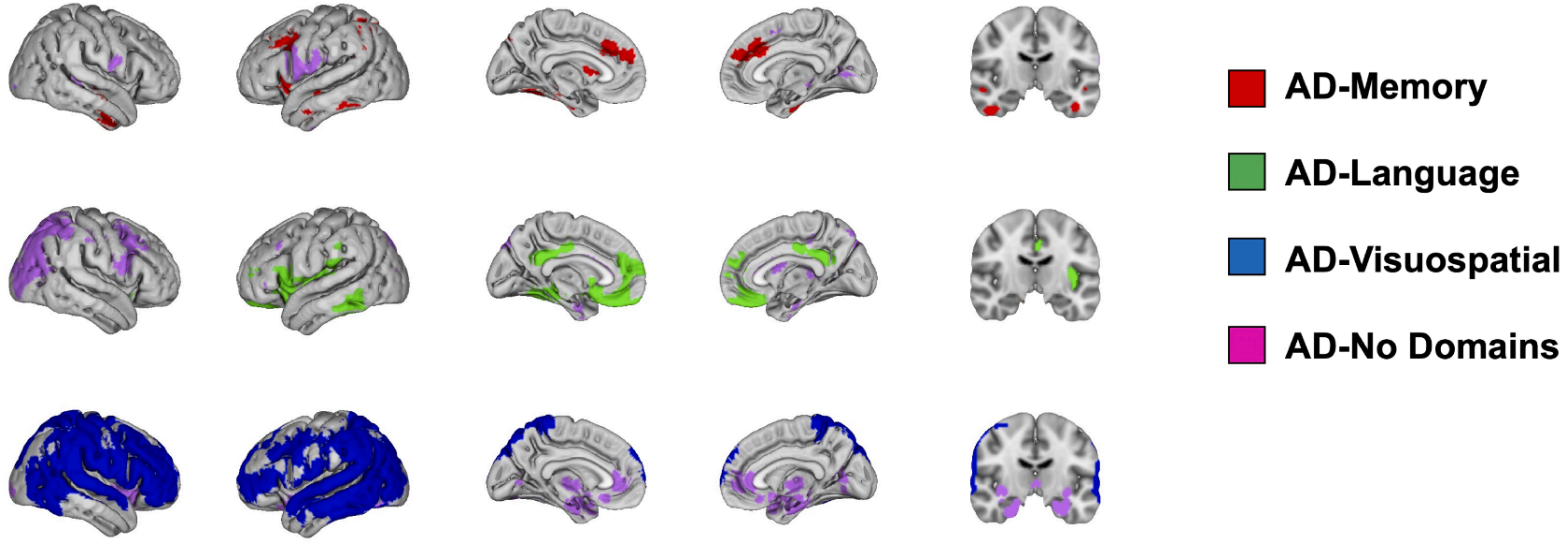
Significant group*time interaction effects from voxel-wise linear mixed model analyses. Significance was set at p<0.001. Red, orange, green and blue colors indicate that that AD-subgroup had faster decline in metabolism compared to AD-No domains and purple indicates that AD-No Domains had faster decline in metabolism compared to the other AD-subgroup.

**Table A1:** Pairwise differences in demographic and clinical characteristics at time of dementia diagnosis. Values depicted are p-values for group differences from pairwise t-tests (continuous variables) and fisher’s exact tests (categorical variables) and Kruskal Wallis test (for non-normally distributed education). Significance = p<0.05 false discovery rate-corrected for the number of group comparisons and bold values fall below this threshold. A – age at time of diagnosis for AD-subgroups and age at first FDG scan for controls, B - MMSE at time of diagnosis for AD-subgroups and MMSE at first FDG scan for controls, C – whole brain FDG SUVR for scan at time of diagnosis (±12 months; n=283) for AD-subgroups and at first FDG scan for controls.

		Control	AD-Memory	AD-Executive	AD-Language	AD-Visuospatial	AD-No Domains
Age^a^	**AD-Memory**	**0.015**					
	**AD-Executive**	0.207	0.595				
	**AD-Language**	**0.000**	**0.005**	0.363			
	**AD-Visuospatial**	0.177	0.810	0.576	**0.009**		
	**AD-No Domains**	**0.000**	0.363	0.803	**0.017**	0.427	
	**AD-Multiple**	0.632	0.576	0.427	**0.014**	0.632	0.363
Sex	**AD-Memory**	0.961					
	**AD-Executive**	0.746	0.863				
	**AD-Language**	0.739	0.746	1.000			
	**AD-Visuospatial**	0.739	0.739	1.000	1.000		
	**AD-No Domains**	1.000	0.961	0.746	0.739	0.739	
	**AD-Multiple**	1.000	1.000	0.840	0.863	0.832	1.000
*APOEε*4, positive	**AD-Memory**	**0.000**					
	**AD-Executive**	**0.030**	0.843				
	**AD-Language**	**0.001**	0.600	1.000			
	**AD-Visuospatial**	**0.000**	0.924	0.924	0.843		
	**AD-No Domains**	**0.000**	0.843	0.924	0.843	1.000	
	**AD-Multiple**	**0.000**	1.000	0.924	0.859	1.000	1.000
Incident AD	**AD-Memory**	-					
	**AD-Executive**	-	1.000				
	**AD-Language**	-	1.000	1.000			
	**AD-Visuospatial**	-	1.000	1.000	1.000		
	**AD-No Domains**	-	1.000	1.000	1.000	1.000	
	**AD-Multiple**	-	1.000	1.000	1.000	1.000	1.000
Education, years	**AD-Memory**	0.131					
	**AD-Executive**	1.000	0.573				
	**AD-Language**	0.131	0.479	0.463			
	**AD-Visuospatial**	0.479	0.718	0.773	0.463		
	**AD-No Domains**	0.068	0.785	0.573	0.573	0.573	
	**AD-Multiple**	1.000	0.463	1.000	0.235	0.640	0.463
Handedness, left	**AD-Memory**	1.000					
	**AD-Executive**	1.000	0.881				
	**AD-Language**	0.839	0.666	1.000			
	**AD-Visuospatial**	0.135	0.135	0.462	0.135		
	**AD-No Domains**	0.135	0.235	0.668	0.135	0.668	
	**AD-Multiple**	1.000	1.000	1.000	0.881	0.666	0.839
MMSE^b^	**AD-Memory**	**0.000**					
	**AD-Executive**	**0.000**	0.958				
	**AD-Language**	**0.000**	0.958	0.958			
	**AD-Visuospatial**	**0.000**	0.958	0.958	0.958		
	**AD-No Domains**	**0.000**	0.072	0.958	0.958	0.691	
	**AD-Multiple**	**0.000**	0.958	0.958	0.958	0.958	0.958
Whole brain FDG SUVR^c^	**AD-Memory**	**0.000**					
	**AD-Executive**	**0.000**	0.095				
	**AD-Language**	**0.000**	0.159	0.643			
	**AD-Visuospatial**	**0.000**	0.747	0.172	0.328		
	**AD-No Domains**	**0.000**	0.509	0.159	0.288	0.986	
	**AD-Multiple**	**0.000**	0.763	0.258	0.479	0.986	0.986

**Table A2:** Number of timepoints available across subjects.

N (%) Timepoints	AD-Subgroups					
	**AD-Memory**	**AD-Executive**	**AD-Language**	**AD-Visuospatial**	**AD-No Domains**	**AD-Multiple**
1	52 (39)	4 (50)	12 (55)	18 (41)	66 (41)	8 (54)
2	29 (22)	1 (13)	4 (18)	9 (21)	38 (24)	1 (7)
3	7 (5)			4 (9)	12 (8)	1 (7)
4	20 (15)	1 (13)	2 (9)	5 (11)	19 (12)	
5	8 (6)	1 (13)	2 (9)	4 (9)	6 (4)	
6	11 (8)	1 (13)		1 (2)	6 (4)	3 (20)
7	6 (4)		1 (5)	1 (2)	8 (5)	1 (7)
8	2 (2)		1 (5)	2 (5)	5 (3)	1 (7)
Total	135	8	22	44	160	15
