# Supplementary material for: Differential trajectories of hypometabolism across cognitively-defined Alzheimer’s disease subgroups
Source: Neuroimage Clin. 2021 Jun 12;31:102725. doi: 10.1016/j.nicl.2021.102725 (PMC8238088; doi:10.1016/j.nicl.2021.102725)
Supplement: Supplementary data 1 [file mmc1.docx]

### Supplemental Text

### Psychometric analyses

What follows is a detailed description of how co-calibration of neuropsychological scores was achieved. First, we will describe how scores from ACT, ADNI, ROS/MAP and PITT were co-calibrated to form the legacy cohort. Then, we will describe the co-calibration of the scores from the ADC cohort to this legacy cohort.

#### Confirmatory factor analyses in each study

Step 1: Domain assignment: In each of the studies (Adult Changes in Thought [ACT], Alzheimer’s Disease Neuroimaging Initiative [ADNI], the Religious Orders Study–Memory and Aging Project (ROS/MAP], and the University of Pittsburgh data set [PITT]), the expert panel (Dr. Trittschuh, Dr. Mez, and Dr. Saykin) assigned items from the neuropsychological battery to one of the four domains (memory, language, executive functioning, and visuospatial ability); other items did not map to any of these domains. The expert panel also assigned each of these items to sub-domains based on the cognitive processes involved in each task. We also noted methods effects where the same stimulus was used in multiple assessments. We also used a data-driven approach looking at patterns of responses among participants to identify alternate possible secondary domain structures.

Step 2: Data quality control: Each of the studies sent their neuropsychological data sets to our team and Ms. Sanders, our data manager, ran an initial quality control on these. Ms. Sanders prepared a data set which included item-level data for individuals at their first Alzheimer’s disease diagnosis. Before running psychometric models, we performed additional recoding of the data. Some items such as Trails A and B were reverse coded. We checked each item to make sure lower values represent lower cognitive performance. We considered the distribution of each item among those with non-missing data and combined categories as needed. Our goals were a.) to avoid sparse categories (operationally defined as <5 responses for each study administering each item) and b.) to have a maximum of 10 categories, which is the maximum number of categories handled by Mplus v7.4. We treated each item as an ordinal indicator of the domain—the numerical value assigned to each category is irrelevant beyond its rank, e.g. calling the lowest category 3 points vs. 18 points makes no difference in how the item is treated or what the final score would be.

We also looked at informative missingness in each study and recoded relevant items accordingly. For example, some of the studies include multiple missing codes, where it was possible to identify refusal to respond to an item as opposed to the interviewer ran out of time and the item was never administered. The first of these—refusal—we took as informative missing and assigned that code to the lowest response category, while the second of these—missing due to scheduling etc.—we took as non-informative missing and omitted that item from consideration.

Step 3: Confirmatory factor analyses: We then turned to confirmatory factor analysis modeling with Mplus using a Robust Weighted Least Squares including terms for the mean and the variance (WLSMV) estimator. We ran four models: a.) a single factor model, with no residual structure; b.) A theory-driven cognitive process bifactor model, using the a priori sub-domain assignments; c.) a theory-driven methods effects bifactor model, using the “methods effects” assignments; and d.) a data-driven bifactor model, using hierarchical clustering-assigned sub-domains. We consulted the expert panel on the sub-domain assignment of items in our data-driven approach to make sure these models made sense to our experts. Our overall strategy was that we would choose the single factor model if adding secondary factors did not markedly improve model fit and if adding secondary factors did not markedly impact any individual’s score (see below).

Our goal with the three bifactor models (models b, c, and d) was to identify a single candidate bifactor model to compare with the single-factor model (model a). Our criteria for selecting the candidate bifactor model included fit statistics (see below) and concordance of model results with theory, such as all loadings on secondary factors being positive. The fit statistics we considered were the confirmatory fit index (CFI) where higher values indicate better fit; thresholds of 0.90 and 0.95 have been used in other settings as criteria for adequate or good fit; the Tucker-Lewis Index (TLI), which has similar criteria as the CFI; and the root mean squared error of approximation (RMSEA), where lower values indicate better fit, and thresholds of 0.08 and 0.05 have been used in other settings as criteria for adequate or good fit.

When comparing the single factor model with the best bifactor model, we a) looked at whether loadings on the primary factor were within 10% of each other across the two models and b) compared the scores for the single factor model vs. scores for the final candidate bifactor model. We used as our threshold a difference of 0.30 units. We chose this value based on the default stopping rule for computerized adaptive testing; this has been used for years as the default level of tolerable measurement differences in the setting of computerized adaptive tests. While arbitrary, this is a level of ambiguity that has been thought to be tolerable in a variety of situations. If there were a substantial number of people for whom the differences in scores were larger than 0.3 from each other, and if the bifactor model conformed to our theory better and had better fit statistics, we selected the final candidate bifactor model as our choice for modeling a domain.

#### Co-calibration of the domains across ACT, ADNI, and ROS/MAP

Step 1: Identification of anchor items: Co-calibration requires either the same people taking different tests or different tests sharing common items. Here we had common items. We identified candidate anchor items with identical content across tests administered in different studies and ensured that their relationship with the underlying ability tested was the same across studies by performing preliminary confirmatory factor analysis models within each study. These items were then used to anchor the scales in each domain to a common metric. We consulted the expert panel (Dr. Trittschuh) to make sure we chose the anchor items correctly.

Step 2: Quality control for anchor items: Anchor items were cleaned and recoded after merging in the items from all the studies making sure that the range of the anchor items were similar in each study. We carefully reviewed documentation from each study to ensure that the stimulus was precisely the same, that the response options were precisely the same or could be re-coded to be the same, and that we were mapping data from each study in a way that the same response would result in the same score regardless of which study the person was enrolled in.

A note regarding response options—in many cases the stimulus is fairly open-ended, such as “can you please draw from memory the figure you copied a while ago”, where the participant is handed a blank sheet of paper and a writing implement. The resulting drawing then gets scored based on how similar it was to the initial stimulus figure. The specific scoring applied to such a stimulus could vary across studies. One study could score such an item as correct vs. incorrect, while another could apply points for various aspects of the drawing. We reviewed the scoring documentation from both studies to determine what “correct” meant in the first study, and how many aspects of the drawing would need to be present for a “correct” score in that study. Then we would map all scores from the second study that would have resulted in a “correct” score in the first study to a “correct” score, and all other scores from the second study to an “incorrect” score. In this way, the resulting score is invariant to which study the person is participating in, as each response would be consistently scored regardless of study.

Step 3: Confirmatory factor analyses: We co-calibrated each of the four domains (memory, executive functioning, language, and visuospatial ability) by incorporating the components of the best model in each study (i.e., the final single-factor or bifactor model selected as described above) into one mega-calibration model.

One particularly tricky aspect of co-calibrating scores using bifactor models is how to handle secondary domains. Some anchor items had loadings on the primary domain (e.g. memory) and also on a secondary domain. That structure by itself does not lead to conceptual problems. However, item representation of the secondary domain may vary across studies, with variable numbers of items, and potential missing data and identifiability issues. To address this we used robust maximum likelihood (MLR) estimation that is robust to missing data, and assigned all subdomain indicators across studies to the same subdomain. Unlike running a CFA model with the WLSMV estimator, a CFA model with MLR estimator does not output fit statistics like CFI/TLI/RMSEA. For our purposes, these secondary domains were nuisances. We performed a number of sensitivity analyses to reassure ourselves that scores on the primary domain were minimally impacted by various ways of specifying the mean and variance on secondary domains. In the final models we selected, we specified a mean of 0 and a variance of 1 for each secondary domain factor, regardless of the number of studies that included items that loaded on that factor.

Once we had fit the final mega-calibration model for each domain, we extracted factor scores for the primary factor (e.g. memory). The resulting scores are on the same metric with a mean of 0 and variance of 1. We used all participants with relevant data to fit data for each domain, so different the scale for each domain was based on models that included different specific people, since some people were missing for some domains. We therefore picked a reference population for standardizing scores for each domain. We used ACT for this, as it was a community-based prospective cohort study, and had a very large sample (n=825) of people with sufficient cognitive data to generate all of our scores. We applied the same standardization to all participants for each study.

Thus, a score of 0, regardless of study, reflects the mean for people with Alzheimer’s disease in the ACT study; and a score of -1, regardless of study, reflects 1 SD below the mean for people with Alzheimer’s disease in the ACT study.

For future data sets in our pipeline such as University of Pittsburgh (PITT) and the Amsterdam Dementia Cohort (ADC), we used these estimated thresholds and loadings of items from the mega-calibration models to obtain scores for individuals. New items (not part of previously co-calibrated studies) were freely estimated while already seen items will have their parameters fixed based on these mega-calibration models.

Confirmatory factor analysis model considerations in co-calibration models

**1.** For all CFA models, we categorized items to ≤ 10 categories. For co-calibration purpose, we had to re-categorize some of the items even though they already had ≤ 10 categories. This was because some studies had more granular data (more categories) for anchor items compared to other studies. In these cases, after we estimated item parameters from the co-calibration model, we re-estimated parameters of the anchor item(s) in the most granular form in the given study. For example, the item “q20mme” was an anchor item for visuospatial ability administered in ACT, ROS/MAP, and ADNI. The item asks individuals to copy intersecting pentagons. In ROS/MAP and ADNI, this item is coded as 0/1 (incorrect/correct) while in ACT it is coded 0–10 (four points for aspects of the left pentagon, four points for aspects of the right pentagon, two points for aspects of the intersection). For co-calibration purpose, we dichotomized this item to 0/1. We consulted scoring algorithms for each of the studies to determine that only scores of 10/10 from the ACT study would have received scores of 1 from ROS/MAP or ADNI; any drawing receiving a score of 9 or fewer from ACT would have scored a 0 in the other studies.

After using re-coded items for co-calibration, we fixed all of the other items to their values from the co-calibration run and freely estimated parameters for re-coded anchors in their most granular form. This approach enabled us to obtain more precise scores in studies that incorporated more granular scoring rules, while still using all items administered across studies to co-calibrate metrics across studies.

**2.** The base co-calibration exercise for each of the four domains was performed across ACT, ROS/MAP, and ADNI. PITT and ADC data were subsequently added with the following steps. For each domain, we identified anchor items and fixed their item parameters to those estimated previously in the base co-calibration models; unique items administered in the new study that were not administered to people in previously co-calibrated studies were freely estimated.

In these models,

a) The mean and variance for the primary factor were freely estimated.

b) If every item in a sub-domain in the new data had parameters available from the co-calibration model, we fixed those item parameters to their previously identified values, and allowed the mean and variance to be freely estimated in the new data.

If no item from a sub-domain had parameters available, then we freely estimated each of the sub-domain loadings, fixing the mean and variance of the subdomain factor to 0 and 1.

If there was a mix of previously specified and new items in a subdomain, we fixed the parameters for the previously specified items, and allowed the mean and variance of the factor and the loadings for new items to be freely estimated in the new data.

**NOTE:** Detailed overview and all code snippets can be obtained from authors on request.

Neuropsychological items by domain for each study and fit statistics from CFA models

MEMORY

ACT: Final model was a theory driven methods-effects bifactor model with CFI = 0.923, TLI = 0.914, and RMSEA = 0.052. The following items were included in the CFA analysis (Supplemental Table 1).

**Supplemental Table 1. Items and secondary structure for memory for the ADNI study**

| **Study** | **Variable** | **Description** | **Secondary Structure** |
| --- | --- | --- | --- |
| ADNI | limmtotal | Logical Memory—Immediate Recall | F1 |
| ADNI | ldeltotal | Logical Memory—Delayed Recall | F1 |
| ADNI | avtot1* | AVLT Trial 1 Total | F2 |
| ADNI | avtot2* | Trial 2 Total | F2 |
| ADNI | avtot3* | Trial 3 Total | F2 |
| ADNI | avtot4* | Trial 4 Total | F2 |
| ADNI | avtot5* | Trial 5 Total | F2 |
| ADNI | avtot6* | Trial 6 Total | F3 |
| ADNI | avtotb* | List B Total | F2 |
| ADNI | avdel30min* | 30 Minute Delay Total | F3 |
| ADNI | avdeltot* | Recognition Score | F4 |
| ADNI | q1score | ADAS Word Recall—score | F2 |
| ADNI | q4score | ADAS Delayed Word Recall | F4 |
| ADNI | q7score | ADAS Orientation—score | F5 |
| ADNI | q8score | ADAS Word Recognition—score |  |
| ADNI | mmdate | What is today's date? | F5 |
| ADNI | mmyear | What is the year? | F5 |
| ADNI | mmmonth | What is the month? | F5 |
| ADNI | mmday | What day of the week is today? | F5 |
| ADNI | mmseason | What season is it? |  |
| ADNI | mmhospit | What is the name of this hospital (clinic, place)? |  |
| ADNI | mmfloor | What floor are we on? |  |
| ADNI | mmcity | What town or city are we in? |  |
| ADNI | mmarea | What county (district, borough, area) are we in? |  |
| ADNI | mmstate | What state are we in? |  |
| ADNI | mmball | Ball | F6 |
| ADNI | mmflag | Flag | F6 |
| ADNI | mmtree | Tree | F6 |
| ADNI | mmballdl | Ball delayed | F7 |
| ADNI | mmflagdl | Flag delayed | F7 |
| ADNI | mmtreedl | Tree delayed | F7 |
| ADNI | imm1sum | Immediate recall of the MoCA list (#1) | F2 |
| ADNI | imm2sum | Immediate recall of the MoCA list(#2) | F2 |
| ADNI | delsum | Delayed recall of the MoCA list |  |

MoCA (blue) items were only administered in ADNI GO/2 while orange items were in all ADNI waves (1/GO/2).

ADNI administered two versions (different word lists) of RAVLT (avtot1–avdeltot) and three different versions of ADAS-Cog items (q*) across waves. We ran the model separately for the two versions. The ADAS-Cog versions were found to be equivalent while the RAVLT versions were not. For determining secondary factor structures and extracting model fit statistics, we considered all RAVLT versions to be equivalent. The different versions of RAVLT were taken into account in the final co-calibration phase.

There were additional MoCA items, which were the same (theoretically) as corresponding items from the Mini-Mental State Examination (MMSE). We excluded MoCA items if those items were already asked as part of the neuropsychological battery.

**Supplemental Table 2. Items and secondary structure for executive functioning for the ADNI study**

| **Study** | **Variable** | **Description** | **Comments** | **Secondary Structure** |
| --- | --- | --- | --- | --- |
| ADNI | clockcirc | Approximately circular face |  |  |
| ADNI | clocksym | Symmetry of number placement |  | F2 |
| ADNI | clocknum | Correctness of numbers |  | F2 |
| ADNI | clockhand | Presence of the two hands |  |  |
| ADNI | clocktime | Presence of the two hands, set to ten after eleven |  |  |
| ADNI | dspanbac | Backward Total Correct |  | F4 |
| ADNI | traascor | Part A Time to Complete |  | F3 |
| ADNI | trabscor | Part B Time to complete |  | F3 |
| ADNI | digitscor | Digit Symbol Total Correct |  | F1 |
| ADNI | dspanfor | Digit Span Forward Total Correct |  | F4 |
| ADNI | q13score | Number cancellation task |  | F1 |
| ADNI | absmeas | Abstraction: watch-ruler |  |  |
| ADNI | abstran | Abstraction: train-bicycle |  |  |
| ADNI | trails | MoCA Trails |  |  |
| ADNI | digback | Digits Backward | 5 trials collapsed |  |
| ADNI | serial | Serial 7 total |  |  |
| ADNI | digfor | Digits Forward |  |  |
| ADNI | letters | List of Letters/Tapping: # Errors |  |  |

**Supplemental Table 3. Items and secondary structure for language for the ADNI study**

| **Study** | **Variable** | **Description** | **Secondary structure** |
| --- | --- | --- | --- |
| ADNI | catanimsc | Category Fluency (Animals) —Total Correct | F1 |
| ADNI | catvegesc | Category Fluency (VegSupplemental Tables) —Total Correct |  |
| ADNI | bnttotal | Total Number Correct (1+3) | F1 |
| ADNI | q2score | ADAS Commands |  |
| ADNI | q5score | ADAS Naming | F1 |
| ADNI | q6score | Ideational Praxis—score |  |
| ADNI | mmwatch | Show wrist watch, ask: What is this? |  |
| ADNI | mmpencil | Show pencil, ask: What is this? |  |
| ADNI | mmrepeat | Say: Repeat after me: no ifs, ands, or buts. |  |
| ADNI | mmhand | Takes paper in right hand |  |
| ADNI | mmfold | Folds paper in half |  |
| ADNI | mmonflr | Puts paper on floor |  |
| ADNI | mmread | Present the piece of paper which reads—CLOSE YOUR EYES—and say: Read this and |  |
| ADNI | mmwrite | Give the participant a blank piece of paper and say: Write a sentence. |  |
| ADNI | camel | Camel |  |
| ADNI | lion | Lion |  |
| ADNI | rhino | Rhinoceros |  |
| ADNI | repeat1 | Repeat Sentence. |  |
| ADNI | repeat2 | Repeat Sentence. |  |
| ADNI | ffluency | Letter Fluency—F: Total number of correct words |  |

**Supplemental Table 4. Items and secondary structure for visuospatial functioning for the ADNI study**

| **Study** | **Variable** | **Description** | **Secondary Structure** |
| --- | --- | --- | --- |
| ADNI | copycirc | Clock copy: Approximately circular face |  |
| ADNI | copysym | Symmetry of number placement |  |
| ADNI | copynum | Correctness of numbers |  |
| ADNI | copyhand | Presence of the two hands |  |
| ADNI | copytime | Presence of the two hands, set to ten after eleven |  |
| ADNI | q3score | Constructional Praxis—score |  |
| ADNI | mmdraw | Present the participant with the Cnstrn Stimulus page. Say: Copy this design |  |

**The ADNI Study (ADNI 1/GO/2)**

ADNI is a longitudinal, multi-site observational study including people with Alzheimer’s disease, people with mild cognitive impairment (MCI), and elderly individuals with normal cognition assessing clinical and cognitive measures, MRI and PET scans (FDG and 11C PIB) and blood and CNS biomarkers. For this study, ADNI contributed data on 607 Alzheimer’s disease cases and 325 healthy controls with Alzheimer’s disease -free status confirmed as of most recent follow-up. Alzheimer’s disease subjects were between the ages of 65–90, had an MMSE score of 20–26 inclusive, met NINCDS/ADRDA criteria for probable Alzheimer’s disease, and had an MRI consistent with the diagnosis of Alzheimer’s disease. Control subjects had MMSE scores between 28 and 30 and a Clinical Dementia Rating of 0 without symptoms of depression, MCI or other dementia and no current use of psychoactive medications. According to the ADNI protocol, subjects were ascertained at regular intervals over 3 years, but for the purpose of our analysis we only used the final ascertainment status to classify case-control status.

Data used in the preparation of this article were obtained from ADNI database (http://adni.loni.ucla.edu). The ADNI was launched in 2003 by the National Institute on Aging (NIA), the National Institute of Biomedical Imaging and Bioengineering (NIBIB), the Food and Drug Administration (FDA), private pharmaceutical companies and non-profit organizations, as a $60 million, 5-year public-private partnership. The primary goal of ADNI has been to test whether serial magnetic resonance imaging (MRI), positron emission tomography (PET), other biological markers, and clinical and neuropsychological assessment can be combined to measure the progression of mild cognitive impairment (MCI) and early Alzheimer’s disease Alzheimer’s disease. Determination of sensitive and specific markers of very early Alzheimer’s disease progression is intended to aid researchers and clinicians to develop new treatments and monitor their effectiveness, as well as lessen the time and cost of clinical trials. The Principal Investigator of this initiative is Michael W. Weiner, MD, VA Medical Center and University of California—San Francisco. ADNI is the result of efforts of many co-investigators from a broad range of academic institutions and private corporations, and subjects have been recruited from over 50 sites across the U.S. and Canada. The initial goal of ADNI was to recruit 800 subjects but ADNI has been followed by ADNI-GO and ADNI-2. To date these three protocols have recruited over 1500 adults, ages 55 to 90, to participate in the research, consisting of cognitively normal older individuals, people with early or late MCI, and people with early Alzheimer’s disease. The follow up duration of each group is specified in the protocols for ADNI-1, ADNI-2 and ADNI-GO. Subjects originally recruited for ADNI-1 and ADNI-GO had the option to be followed in ADNI-2. For up-to-date information, see www.adni-info.org.
